# Polyanionic carbosilane dendrimers as a new adjuvant in combination with latency reversal agents for HIV treatment

**DOI:** 10.1186/s12951-019-0500-4

**Published:** 2019-05-21

**Authors:** Ignacio Relaño-Rodríguez, Raquel Juárez-Sánchez, Carolina Pavicic, Eduardo Muñoz, Maria Ángeles Muñoz-Fernández

**Affiliations:** 1 Molecular Immunology Laboratory, Hospital General Universitario Gregorio Marañón, Madrid, Spain; 2 Health Research Institute Gregorio Marañón (IiSGM), Spanish HIV HGM BioBank, Madrid, Spain; 3 Networking Research Center on Bioengineering, Biomaterials and Nanomedicine (CIBER-BBN), Madrid, Spain; 4 Innohealth, Parque Científico de Madrid, Madrid, Spain; 5 Department of Cell Biology, Physiology and Immunology, Instituto Maimónides de Investigaciones Biomédicas de Córdoba (IMIBIC)/Reina Sofia University Hospital University of Córdoba, Córdoba, Spain

**Keywords:** Nanomedicine, Dendrimers, HIV-1 latency, Latency reversal agents

## Abstract

**Background:**

The major obstacle impeding human immunodeficiency virus-1 (HIV-1) eradication in antiretroviral treatment (ART) treated HIV-1 subjects is the establishment of long-lived latently infected resting CD4^+^ T cells. Due to the fact that no drug has been effective, the search for new drugs and combinations are a priority in the HIV cure. Treatments based on nanotechnology have emerged as an innovative and promising alternative to current and conventional therapies. In this respect, nanotechnology opens up a new door for eliminating latent HIV infection. We studied the role of G1-S4, G2-S16 and G3-S16 polyanionic carbosilane dendrimers in the context of latent HIV-1 persistence. Moreover, we study the efficiency of these dendrimers in combination with latency reversal agents (LRAs) against HIV-1 infection.

**Methods:**

J89GFP lymphocyte and THP89GFP monocyte derived cell lines latently infected with HIV-1 p89GFP were used as an in vitro model of latency for our study. Viability assays by 3-(4-5-dimethylthiazol-2-yl)-2,5-diphenyltetrazolium bromide (MTT) and lactate dehydrogenase (LDH) were performed to determine the working concentrations of dendrimers and LRAs. Both cell lines were treated with G1-S4, G2-S16 and G3-S16 either alone or in combination with bryostatin (BRY), romidepsin (RMD) or panobinostat (PNB) for 24 and 48 h. The expression pattern of GFP was measured by flow cytometry and referred as measure of viral reactivation.

**Results and discussion:**

The combination treatment of the dendrimers with the protein kinase C (PKC) agonist did not modify the antilatency activity in J89GFP lymphocyte cell line. Interestingly enough, G3-S16 dendrimer alone and its combination with BRY, RMD or PNB showed a significant increased expression of GFP in the THP89GFP monocyte cell line.

**Conclusion:**

We showed for the first time that nanoparticles, in this case, G3-S16 anionic carbosilan dendrimer may play an important role in new treatments against HIV-1 infection.
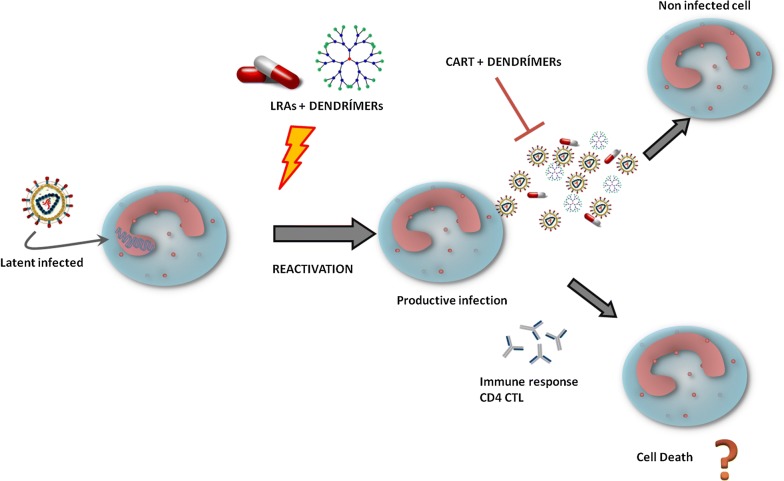

## Background

Human immunodeficiency virus-1 infection can be treated effectively in the developed world, using new combinations of antiretroviral treatments (cARTs). Despite prolonged cARTs, the persistence of HIV-1 in resting CD4^+^ T cells reservoirs harbouring transcriptional silence and replication-competent proviruses presents the major hurdle to HIV-1 eradication. These latently-infected cells are a permanent source for virus reactivation and lead to a rebound of the viral load after interruption of cARTs [1, 2].

Various therapeutic interventions to eradicate HIV-1 focus on the stimulation of HIV-1 production from latently infected cells. These interventions involve the use of latency-reversing agents (LRAs) such as prostratin (PST), bryostatin-1,2 (BRY), panobinostat (PNB), and romidepsin (RMD) [3–9]. LRAs reactivate latently-infected cells, whereas the cARTs prevent spreading HIV-1 infection. It has been published that BRY, PNB and RMD present a good reactivation activity both in vitro and ex vivo and have currently been used in HIV-1 eradication clinical trials [10–12]. However, a decrease of the viral reservoir or total eradication of HIV-1 in infected subjects has not been achieved.

There are two major problems in HIV-1 infection: (1) virus persistence in reservoirs in a latent form integrated into the host genome, and virus replication when cells undergo activation; (2) inhibition of current ARV drugs with retrotranscription or with HIV-1 protease. This strategy presents additional troublesome such as the appearance of resistance. Drugs that interfere with ARV entry such as enfurtivide, which inhibits viral and cell membrane fusion, have been postulated as a possible alternative. However, the first cases of resistance have already been described [13].

In the last decade, the nanotechnology has been improved by the development and discovery of a wide range of novel nanoparticles. These new nanotechnology applications are easy to design, develop and synthesize. Dendrimers are promising nanoparticles, described as highly branched tree-like molecules between 1 and 40 nm [14]. We work with G1-S4, G2-S16 and G3-S16 polyanionic carbosilane dendrimers in the context of the HIV-1 infection [15, 16].

Our objective is to study the potential use of our polyanionic carbosilane dendrimers that will be applied in the “shock and kill” therapy increasing the HIV-1 reactivation and avoiding new HIV-1 infections.

## Materials and methods

### Dendrimers

**Fig. 1:**
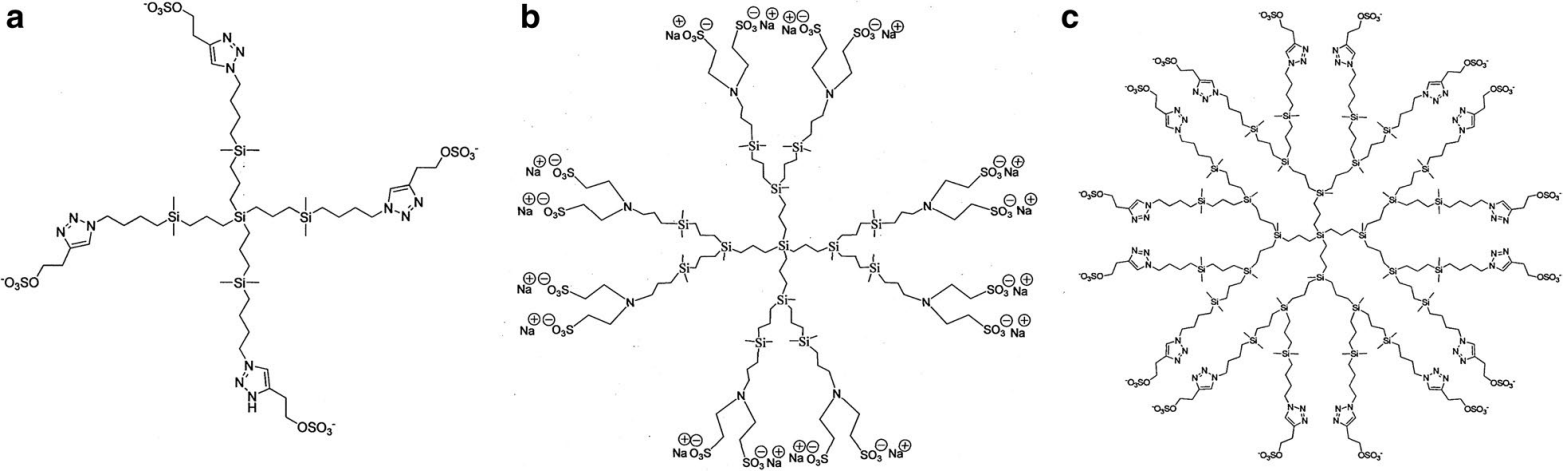
Molecular representation of dendrimers. **a** G1-S4 with 4 sulfate end groups, **b** G2-S16 with 16 sulfonate end groups and **c** G3-S16 with 16 sulfate end groups were represented. The generation of dendrimers is determined by considering that each generation corresponds to the number of repeating layers of silicon atoms

Polyanionic carbosilane dendrimers G1-S4 with 4 sulfate groups in periphery, G2-S16 with 16 sulfonate groups in the periphery, and G3-S16 with 16 sulfate groups in the periphery were synthesized and analyzed according to methods reported by the Dendrimers for Biomedical Applications Group of University of Alcalá (Madrid, Spain) [15, 16]. Stock solution of dendrimers (10 mM) and subsequent dilutions to working concentrations were prepared in nuclease-free water (Promega, Madrid, Spain). The schematic structures of the polyanionic carbosilane dendrimers are presented in Fig. 1.

### Zeta potential

These measurements were done in a Zetasizer Nano ZS (Malvern Instruments Ltd., UK) at 25 °C using disposable Malvern plastic cuvettes (1 ml), by solving 1 mg of dendrimers G1-S4, G2-S16 and G3-S16 in purified water, which was previously filtered through 0.22 µm filter.

### Cell lines

J89GFP lymphocyte and THP89GFP monocyte cell lines (kindly donated by Dr. David N Levy, NYU, USA), are derived cell lines latently infected with recombinant HIV-1 p89 GFP. J89GFP cell line was maintained in RPMI complemented with 5% FBS, 125 mg/ml ampicillin, 125 mg/ml cloxacillin and 40 mg/ml gentamicin (Normon, Madrid, Spain). THP89GFP cell line was maintained in ultra-low attachment culture plates in DMEM complemented with 5% FBS, 125 mg/ml ampicillin, 125 mg/ml cloxacillin and 40 mg/ml gentamicin (Normon, Madrid, Spain) and were maintained according to the protocol described by Kutsch et al. [17].

### Reagents

Bryostatin-1,2 (BRY) and prostratin (PST) were obtained from Sigma-Aldrich (St. Louis, MO, USA), panobinostat (PNB) and romidepsin (RMD) were obtained from Selleck Chemicals (Houston, TX). Drugs were dissolved in dimethyl sulfoxide (DMSO) (Sigma-Aldrich, St. Louis, MO, USA) to prepare stock solutions. DMSO concentration in cell cultures was lower than 0.001%.

### Mitochondrial activity assay

The mitochondrial toxicity of compound concentrations was tested by the 3-(4-5-dimethylthiazol-2-yl)-2,5-diphenyltetrazolium bromide (MTT) assay (Sigma, St Louis, USA) according to manufacturer’s instructions in G1-S4, G2-S16 and G3-S16 dendrimers for 24 and 48 h and BRY, PST, PNB or RMD for 24 h. Briefly, 6 × 10^4^ of J89GFP or 2.5 × 10^4^ THP89GFP cells were seeded in 96-well plates and treated with the desired compound for 24 or 48 h. After incubation period, culture medium was discarded and 220 μl of a 1:11 MTT/OptiMEM solution was added to the cultured cells. After 3 h, the supernatant was removed, and formazan crystals were dissolved in 200 μl DMSO (Sigma, St. Louis, MO, USA). All points were performed in triplicate. DMSO 10% was used as death cellular control. The concentration range of each compound examined in this study is in agreement with previously published results [18, 19].

### Membrane integrity assay

Cell membrane integrity was measured by the lactate deshidrogenase (LDH) assay CytoTox 96^®^ Non-Radioactive Cytotoxicity (Promega, Spain, Madrid) following manufacturer’s instructions. Briefly, 3 × 10^4^ cells were seeded in 96-well plates and treated with the desired compounds and their combinations for 24 h and 48 h. After incubation period, cells were lysed in 0.9% Triton X-100 (Promega, Spain, Madrid) for 45 min at 37 °C. After incubation, 50 μl of LDH reagent (Promega, Spain, Madrid) was added for 30 min at room temperature, protected from light absorbance was read in a Berthold Plate Reader at 490 nm. All points were performed by triplicate.

### Confocal microscopy

GFP expression and LIVE/DEAD cells were analysed by confocal microscopy. J89GFP and THP89GFP were seeded at a density of 3 × 10^5^ in 24-well culture plates and ultra-low attachment plates, respectively. Subsequently, J89GFP and THP89GFP were treated with BRY, G2-S16 or G3-S16 for 48 h at 37 °C. After incubation, both cell lines were treated with NUCLEAR-ID^**®**^ Blue/Red cell viability reagent (ENZO, Farmingdale, New York) following manufacturer’s instructions and visualized in a Leica TSC SPE confocal microscope. All points were performed in duplicate. DMSO 10% was used as death cellular control.

### Latent HIV reactivation

GFP-fluorescence pattern measured by flow cytometry was used to determine viral reactivation in J89GFP and THP89GFP cell lines. J89GFP and THP89GFP cells were seeded at a density of 4 × 10^5^ in 24-well culture plates and ultra-low attachment 24-well culture plates respectively, and after were stimulated with the indicated compounds. At least 30,000 cells were analysed by flow cytometry. The integrated mean fluorescence intensity (iMFI, percentage of GFP expressing cells *MFI) of live cells was used as a measure of HIV-1 reactivation.

### Statistics

Statistical analysis was performed using GraphPad software Prism v.5.0 (GraphPad Software, San Diego, CA USA) between two groups (control versus different dosages of compounds or LRA-treated versus combined LRAs and G1-S4, G2-S16 or G3-S16) were assessed by using a paired t-test. (*p < 0.05; **p < 0.005; ***p < 0.001).

## Results and discussion

### Biocompatibility of latency reversing agents and dendrimers

We previously described the potent activity of G1-S4, G2-S16 and G3-S16 dendrimers against HIV-1 infection [20–22]. Cell viability of sulfonate G2-S16 or sulfate G1-S4 and G3-S16 dendrimers, of nanoscale between 1 and 20 nm diameter, versatility and multi branching properties were studied on J89GFP lymphocyte and THP89GFP monocyte cell lines by MTT assay. Moreover, we studied by MTT and LDH assays the viability of BRY, PNB, PST and RMD in J89GFP and THP89GFP cell lines. The combinations of LRAs and dendrimers were analyzed by LDH.

**Fig. 2:**
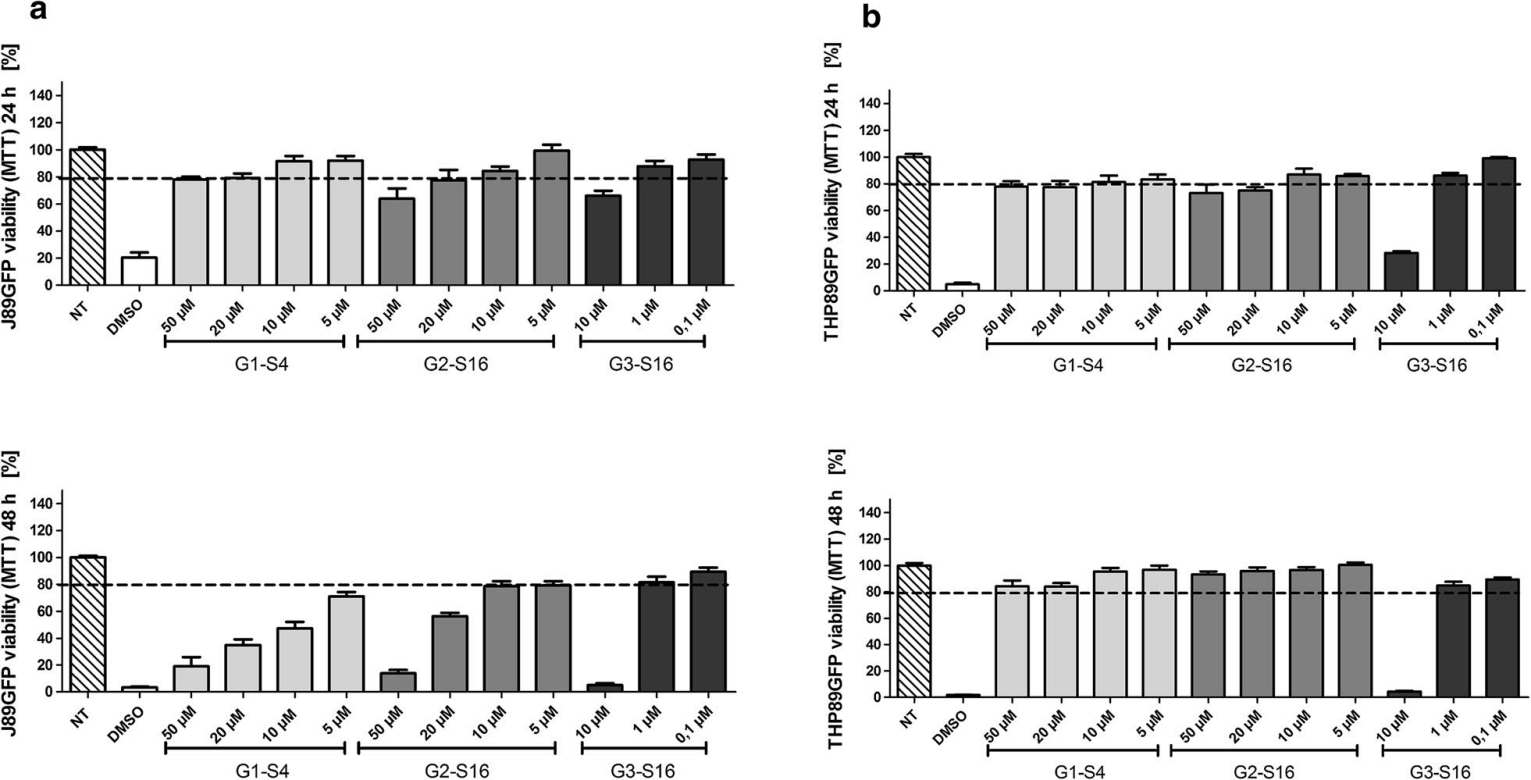
Mitochondrial activity studies of polyanionic carbosilane dendrimers. MTT assays were performed in **a** J89GFP lymphocyte and **b** THP89GFP monocyte cell lines for 24 h and 48 h. Cell lines were treated with increased concentration range (0.2–50 μM) of G1-S4, G2-S16 or G3-S16 dendrimers. Non-treated (NT) cells were used as cell viability control. DMSO was used as cell death control. *NT* non-treated control, *DMSO* dimethyl sulfoxide. The mean values (mean ± SD) of two or three independent experiments are shown (*p < 0.05; **p < 0.005; ***p < 0.001)

J89GFP and THP89GFP cell lines were seeded and treated with G1-S4, G2-S16 or G3-S16 dendrimer in various range of concentrations to determine the maximum non-toxic concentration (Fig. 2). Dendrimers were considered non-toxic when the survival rate was > 80%. Non-treated (NT) cells were used as cell viability control and DMSO 10% was used as death cellular control for MTT assays.

The results of MTT showed that G1-S4 dendrimer was not toxic at 50 µM and G2-S16 at 20 µM in J89GFP cell line and both dendrimers were non-toxic at 50 µM in THP89GFP cell lines during 24 h treatment. G1-S4 and G2-S16 dendrimers did not produce toxicity up to 20 µM in THP89GFP and 10 µM in J89GFP after 48 h. However, G3-S16 maximum non-toxic concentration was only 1 µM in both cell lines. G3-S16 was the dendrimer with the highest toxicity rates due to its major generation and bigger size. G2-S16 dendrimer with sulfonate groups in the surface was less toxic than G1-S4 with sulfate groups in J89GFP cell line after 48 h treatments, showing that not only the generation and size of the dendrimers, but also the functionalizing molecules are determinants for the biocompatibility.

**Fig. 3:**
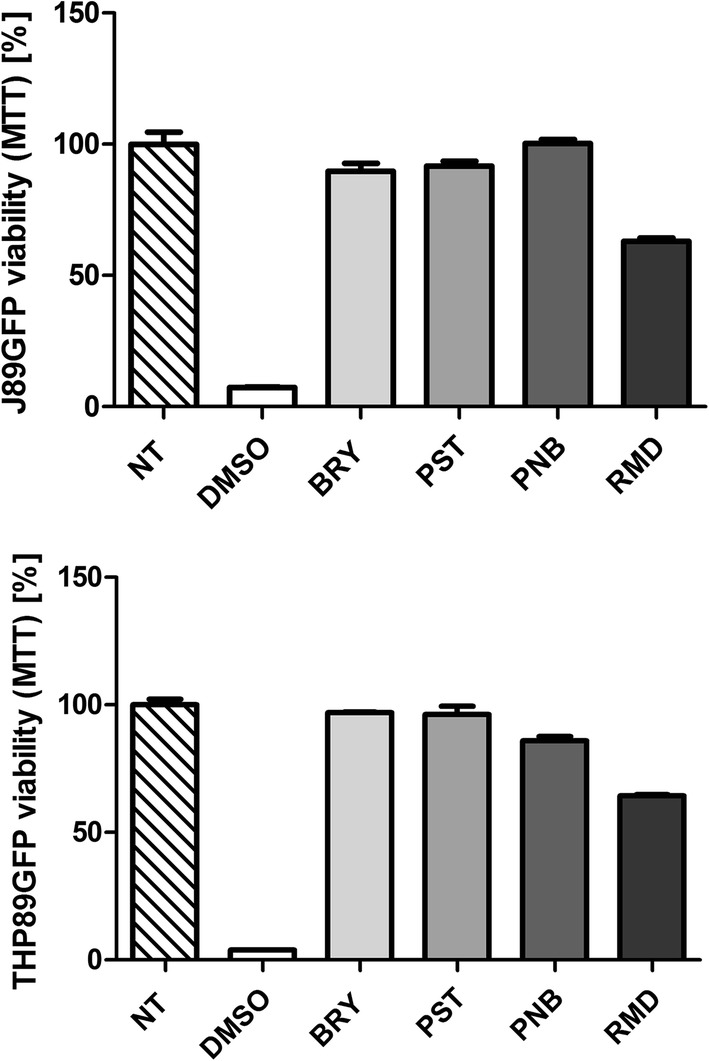
Mitochondrial activity studies of latency reversal agents. MTT assays were performed on J89GFP lymphocyte and THP89GFP monocyte cell lines during 24 h. Both cell lines were treated with BRY up to 100 nM, PST up to 20 µM, PNB up to 40 nM, and RMD up to 20 nM. Non-treated (NT) cells were used as cell viability control. DMSO was used as cell death control. *NT* non-treated control, *DMSO* dimethyl sulfoxide. The mean values (mean ± SD) of three independent experiments are shown (*p < 0.05; **p < 0.005; ***p < 0.001)

The selection of the LRA concentrations was performed taking into account the maximum non-toxic concentration for each drug previously reported [23, 24]. BRY was non-toxic up to 100 nM, PST up to 20 µM, PNB up to 40 nM, and RMD showed toxicity at 20 nM (Fig. 3).

**Fig. 4:**
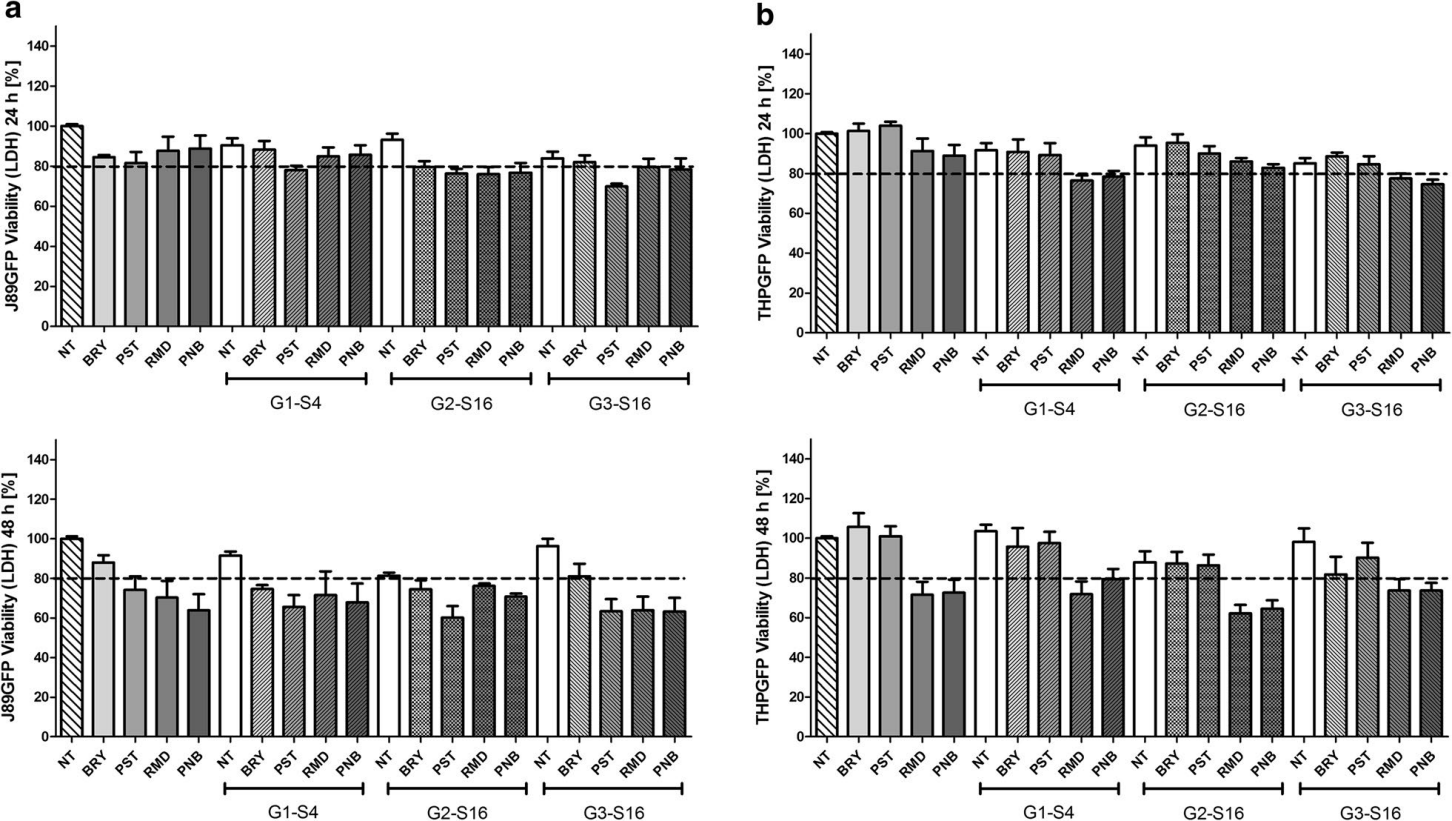
Membrane integrity studies of carbosilane dendrimers and LRAs combinations. LDH assays were performed on **a** J89GFP lymphocyte and **b** THP89GFP monocyte cell lines during 24 h and 48 h. Cell lines were treated with G1-S4 (10 µM), G2-S16 (10 µM) or G3-S16 (1 µM) dendrimers. Both cell lines were treated with BRY (100 nM), PST (20 µM), PNB (40 nM) or RMD (20 nM). Non-treated (NT) cells were used as cell viability control. *NT* non-treated control, *BRY* bryostatin, *PST* prostratin, *RMD* romidepsin, *PNB* panobinostat. The mean values (mean ± SD) of three independent experiments are shown (*p < 0.05; **p < 0.005; ***p < 0.001)

Combinations of dendrimers with LRAs in the J89GFP cell line showed no toxicity at 24 h. The viability decreased below 80% for PST, RMD and PNB after 48 h treatment analysed by LDH assay. Results indicated that after 48 h dendrimers did not produce cytotoxicity. However, combinations with LRAs reduced viability, probably due to the basal toxicity of LRAs on this cell line (Fig. 4).

In THP89GFP cell line, LRAs treatment for 24 h did not produce a decrease in the cell viability. Similarly, the dendrimers alone or in combination with BRY and PST did not produce toxicity. On the other hand, combination of G1-S4 and G3-S16 with RMD and PNB caused a 20% decrease in viability. No cell toxicity was observed at 48 h following single treatment with either BRY, PST or dendrimers single treatment. However, RMD, PNB and their combinations with dendrimers reduced viability below 80% in THP89GFP cells.

Exposure of J89GFP or THP89GFP cells to either G2-S16 (up to 10 µM) or G3-S16 (up to 1 µM) did not show toxicity following 24 and 48 h of treatment. However, G1-S4 reduced J89GFP cells viability measured by MTT at 48 h.

Potential z measurements were performed to study the aggregation ratio in aqueous solution. Due to technical issues with the small size of G1-S4, potential z for this dendrimer was not available. On the other hand, G2-S16 dendrimer z potential was − 74.0 mV showing high long-term stability in aqueous solutions which demonstrate a good biocompatibility in cell culture. G3-S16 showed a higher z potential, − 46.2 mV. These results agree with the viability data obtained by MTT and LDH assays, indicating that just 1 µM was the maximum non-toxic concentration for G3-S16, while for G2-S16 was ten times higher, 10 µM.

**Fig. 5:**
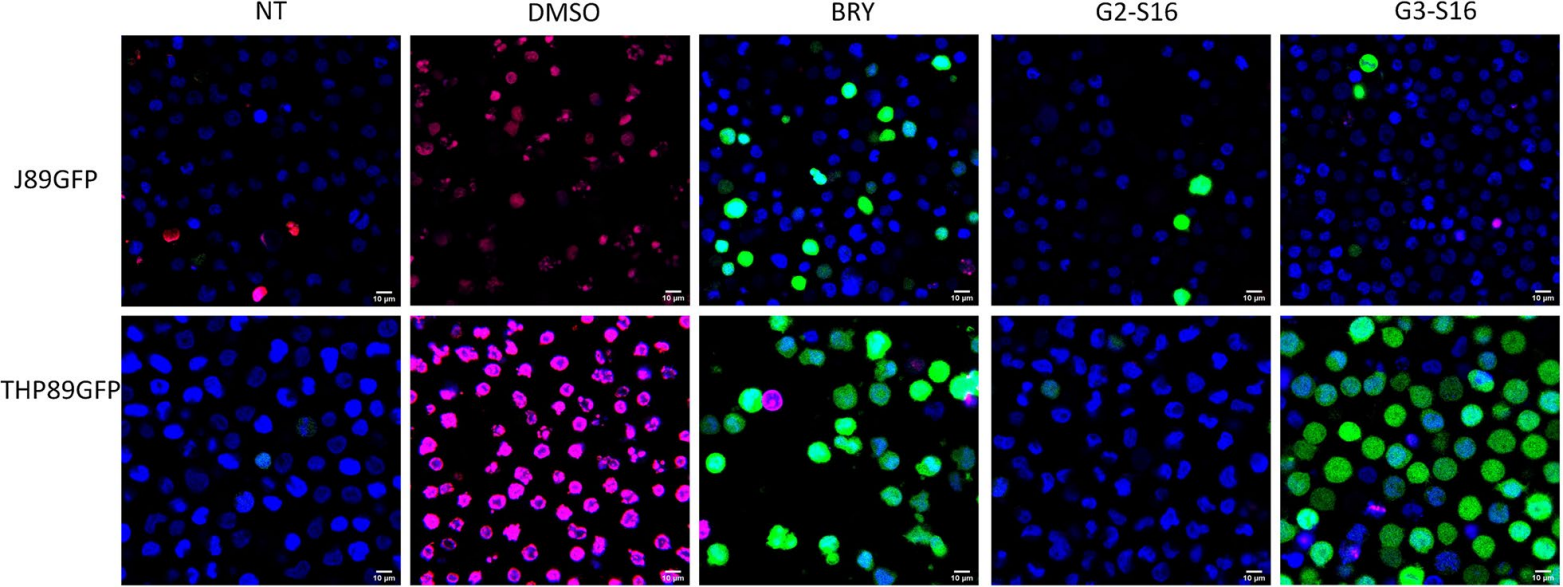
Confocal microscope images of reactivation and LIVE/DEAD cells. J89GFP or THP89GFP cell lines were treated with BRY (100 nM), G2-S16 (10 µM) or G3-S16 (1 µM) for 48 h. After incubation, cells were stained with NUCLEAR-ID^®^ Blue/Red. Blue: live cells. Green: HIV-1 reactivation. Red: cellular death. Non-treated (NT) cells were used as cell viability control. DMSO was used as cell death control. *NT* non-treated control; *DMSO* dimethyl sulfoxide; *BRY* bryostatin. Representative images of two independent experiments performed in duplicate are shown

Reactivation was measured by determining GFP expression in living cells. Confocal microscope images of non-treated, G2-S16, G3-S16, BRY and DMSO 10% were taken to confirm the expression of GFP in live cells (Fig. 5). Results of NT and DMSO 10% in both cell lines represented viability and death control, showed in blue and red respectively. BRY was used as reactivation control, represented in green. In J89GFP cell line results showed that only BRY treatment leads to HIV-1 reactivation. However, in THP89GFP cell line, results indicate that G3-S16 dendrimer promotes GFP expression.

### Reactivation profile of LRAs in combinations with dendrimers in latently HIV-1 infected cell lines

Our dendrimers are directed for a possible therapeutic treatment. In this context we have previously shown that these dendrimers inhibit HIV-1 infection and can be used in combination with different antiretrovirals [25–27]. However, we do not know what function the dendrimers have in the presence of LRAs. Therefore, we studied their potential effect in the presence of LRAs. To determine the viral reactivation, the GFP-fluorescence pattern was measured by flow cytometry.

**Fig. 6:**
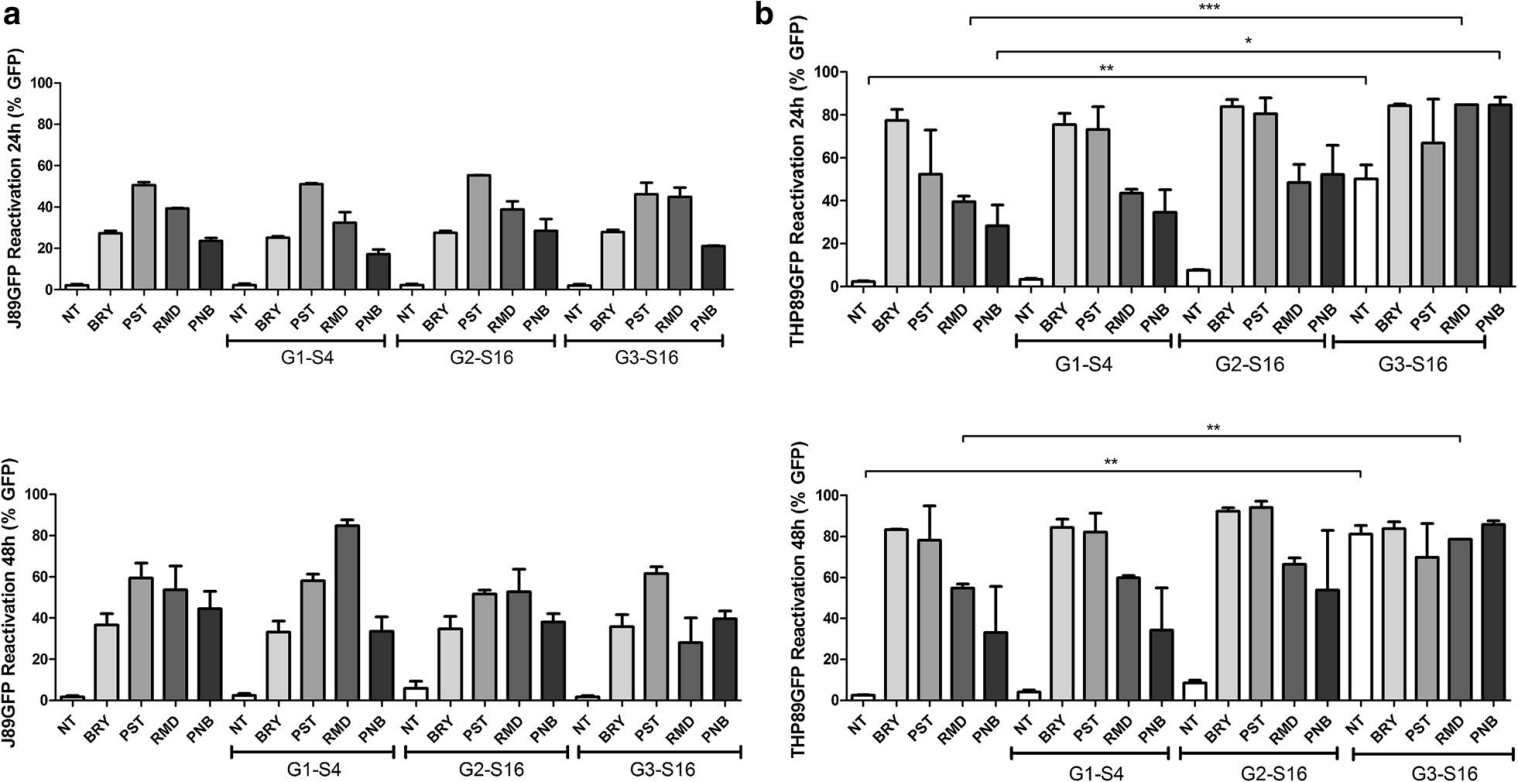
Reactivation profile of BRY, PST, RMD and PNB in combination with G1-S4 (10 µM), G2-S16 (10 µM), G3-S16 (1 µM) dendrimers. J89GFP (**a**) and THP89GFP (**b**) cell lines were treated with BRY (100 nM), PST (20 µM), RMD (20 nM) and PNB (40 nm), either alone or in combinations with dendrimers for 24 h and 48 h. The integrated mean fluorescence intensity (iMFI, percentage of GFP expressing cells *MFI) of live cells was used as a measure of HIV-1 reactivation. *NT* non-treated control, *BRY* bryostatin, *PST* prostratin, *RMD* romidepsin; *PNB* panobinostat. The mean values (mean ± SD) of two or three independent experiments are shown (*p < 0.05; **p < 0.005; ***p < 0.001)

The HIV-1 reactivation effect of BRY, PST, PNB and RMD were analysed as individual drugs or in combination with G1-S4, G2-S16 or G3-S16 dendrimers at various ratios. The concentrations of the dendrimers were based on the non-toxic concentration 10 µM G1-S4, 10 µM G2-S16 and 1 µM G3-S16 previously selected. The selection of LRA concentrations were performed, taking into account the maximum non-toxic concentration of each LRA based on the scientific literature, 100 nM BRY, 20 µM PST, 40 nM PNB, and 20 nM RMD. After 24 h and 48 h of exposure, the reactivation effect was measured by flow cytometry and expressed as GFP (integrated MFI or iMFI) (Fig. 6).

Our results indicate the enhanced GFP expression with the treatment of the LRAs alone in J89GFP cell line at 24 h of exposure. The PST induced the highest response at 55% of EGFP expression. In the case of the dendrimers alone, the GFP expression was not modified in regards of the non-treated control. The combination treatment of LRAs and dendrimers indicate that the antilatency activity of the LRAs was not modified, even in some cases it tends to increase slightly in combination with G1-S4, G2-S16 or G3-S16 dendrimers. At 48 h BRY, PST, RMD or PNB tend to increase the GFP expression in J89GFP cell line. Nevertheless, RMD in combination with G1-S4 showed an increased tendency in the expression of GFP. Neither of the three dendrimers studied alone show any variation in the GFP expression in the lymphocytic derived cell line. The combination of either G1-S4, G2-S16 or G3-S16 with BRY and PST in J89GFP lymphocytic cell line at 48 h did not modify the GFP expression. Our data indicate that the combination treatment of our dendrimers with the PKC agonist did not modify the antilatency activity.

In the THP89GFP, monocytic derived cell line, we obtained different results. The GFP expression in the single LRAs treatment at 24 h was greatly increased in comparison with the J89GFP lymphocytic cell line, reaching 80% of GFP expression with BRY. The LRAs combination with G1-S4 or G2-S16 dendrimer did not show any variation of the GFP expression in regards of treatment of LRAs alone. Surprisingly, in THP89GFP monocytic cell line, the G3-S16 dendrimer alone treatment showed a significant increased expression of GFP reaching 50%. We hypothesize that high size of G3-S16 in addition to sulfate groups in its periphery activate the monocytes, thus unleashing transcription factors such as NF-Kb or Sp1, forcing the viral reactivation and transcription [28]. In the case of the combinations of LRAs with G3-S16, the results showed an enhanced expression of GFP reaching almost 80% for BRY, RMD and PNB. This result proves a new possible approach for a combination treatment in the “shock and kill” method with dendrimers, which not only inhibit the entry of new replicative viruses, but also help with the reactivation activity of LRAs. Although our results are promising, the mechanism of our dendrimers reactivation and the use of more physiological models such as latently infected primary cells or in vivo models should be studied. Summing up, we demonstrated for the first time that nanoparticles, in this case dendrimers may play an important role in new treatments against HIV-1 infection.

## Data Availability

The datasets used and/or analysed during the current study are available from the corresponding author on reasonable request.
